# The effects of glucagon-like peptide-1 receptor agonists on sympathetic neuron activity

**DOI:** 10.1038/s41440-026-02633-5

**Published:** 2026-04-10

**Authors:** Yui Koyanagi, Kamon Iigaya, Keiko Ikeda, Hiroshi Onimaru, Masahiko Izumizaki

**Affiliations:** 1 Department of Physiology, Showa Medical University School of Medicine, Tokyo, 142-8555 Japan; 2 Deptartment of Oral Physiology, Showa Medical University School of Dentistry, Tokyo, 142-8555 Japan

**Keywords:** Glucagon-like peptide-1, Exendin-4, Sympathetic nerve activity, Rostral ventrolateral medulla, Drug-induced tachycardia

## Abstract

Glucagon-like peptide-1 (GLP-1) receptor agonists are widely used to manage type 2 diabetes mellitus. However, there are reports indicating that patients administered GLP-1 receptor agonists often experience an increased heart rate. Although activation of the sympathetic nervous system may be involved in this response, the detailed mechanisms of action of GLP-1 receptor agonists are still not well understood. We hypothesized that GLP-1 receptor agonists could excite sympathetic nerve activity through direct effects on sympathetic-related neurons in the spinal cord and the medulla oblongata. Therefore, we examined the effects of a major GLP-1 receptor agonist, exendin-4, on sympathetic nerve activity at three different levels using in vitro preparations: (1) sympathetic nerve activity from the sympathetic nerve trunk, (2) preganglionic neurons in the intermediolateral cell column at the Th2–4 level of the spinal cord and (3) neurons in the rostral ventrolateral medulla corresponding to the C1 pressor area. Brainstem-spinal cord preparations were isolated from newborn rats (P0-P4) under deep isoflurane anesthesia and superfused with artificial cerebrospinal fluid, bubbled with 95% O_2_ and 5% CO_2_ at 25–26 °C. We found that 20–100 nM exendin-4 induced an increase in sympathetic nerve activity and the effect was blocked by the application of a GLP-1 antagonist. The application of 100 nM exendin-4 also induced membrane depolarization of the intermediolateral cell column and rostral ventrolateral medulla neurons. These results suggested that exendin-4 could induce increased sympathetic nerve activity via excitation of sympathetic-related neurons in the medulla and spinal cord.

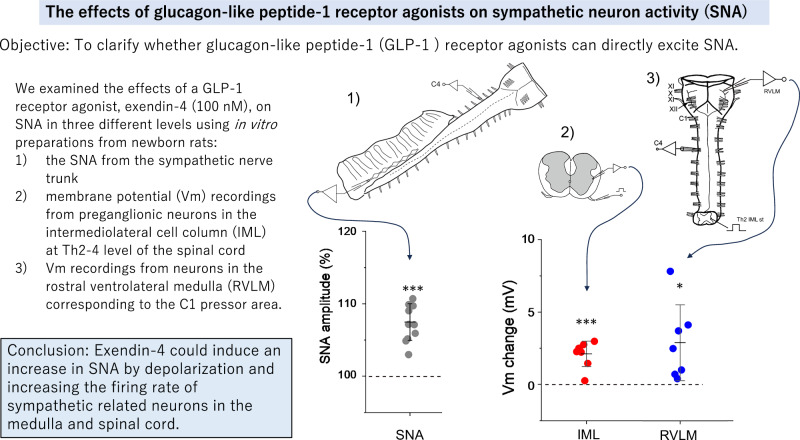

## Introduction

Glucagon-like-peptide 1 (GLP-1) is a neuropeptide secreted by enteroendocrine L cells in the small intestine. It acts as an incretin and exerts a variety of physiological and pharmacological effects such as the promotion of insulin secretion [1]. GLP-1 is also produced by preproglucagon neurons in several brain regions including the nucleus tractus solitarius (NTS), and is involved in various brain functions such as the regulation of food intake and sympathetic nerve activity (SNA) [2–5]. The physiological properties of GLP-1 receptor agonists have led to their development as a class of medications that effectively reduce HbA1c levels and promote body weight loss, thereby offering a significant advantage in the comprehensive management of type 2 diabetes mellitus as reviewed by Rolek et al. [6]. or Masuda et al. [7]. GLP-1 receptor agonists are also recognized as novel drugs that reduce the incidence of cardiovascular events such as myocardial infarction, a common underlying cause of heart failure [8, 9]. Thus, it is a recent trend for diabetic patients with heart failure to receive GLP-1 receptor agonists [10]. However, there are reports that patients frequently experience an increase of heart rate after using GLP-1 receptor agonists [11]. Several previous studies have shown that GLP-1 receptor agonists cause tachycardia in animals [12, 13] and humans [14]. Moreover, Jorsal et al. [15] demonstrated that liraglutide, a GLP-1 receptor agonist, increased serious adverse events such as ventricular tachycardia and atrial fibrillation in patients with stable chronic heart failure.

Yamamoto et al. [12] reported that GLP-1 agonists dose-dependently increased blood pressure and heart rate in rats probably via activation of neurons in autonomic control sites in the brain, including medullary catecholamine neurons that provide input to sympathetic preganglionic neurons. Holt et al. [2] showed that the application of GLP-1 receptor agonists increased blood pressure and heart rate, and suggested that GLP-1 agonists could increase cardiac sympathetic preganglionic neuron activity in mice. In contrast, Oshima et al. [16] showed that presympathetic neurons in the rostral ventrolateral medulla (RVLM) of newborn rats were hyperpolarized during the application of GLP-1 peptide. They suggested that GLP-1 could cause a decrease in blood pressure. Thus, the effects of GLP-1 agonists on SNA and the pathways of action remain to be clarified.

In the present study, we aimed to reveal how GLP-1 receptor agonists could affect the sympathetic nervous system. For this purpose, we used in vitro preparations from newborn rats [16–18] and exendin-4 (also known as exenatide), a GLP-1 agonist that has been most commonly used in animal experiments. We investigated the effects of exendin-4 on (1) sympathetic nerve output, (2) preganglionic neurons (and interneurons) in the intermediate lateral cell column (IML) of the thoracic cord, and (3) neurons in the RVLM, including presympathetic neurons.

## Methods

### Preparations

The experimental protocols were approved by the Institutional Animal Care and Use Committee of Showa University (approval no. 124046).

Experiments were performed using brainstem-spinal cord preparations (or spinal cord block preparations) from newborn Wistar rats (age: 0–4 days, either sex). Rats were deeply anesthetized with isoflurane, and the brainstem and/or spinal cord were isolated and placed in a 2 ml experimental chamber. The brainstem was rostrally cut at a level just rostral to the anterior inferior cerebellar artery. Preparations were superfused at a rate of 3.0 ml/min with the following artificial cerebrospinal fluid (ACSF) [19] (in mM): 124 NaCl, 5.0 KCl, 1.2 KH_2_PO_4_, 2.4 CaCl_2_, 1.3 MgCl_2_, 26 NaHCO_3_ and 30 glucose, equilibrated with 95% O_2_ and 5% CO_2_, pH 7.4, at 26–27 °C.

To evaluate the effects of the drug on different levels of neuronal activity involved in sympathetic nerve outputs, we performed experiments using the following three types of in vitro preparations:

1. Sympathetic nerve retained preparation: SNA was recorded in preparations with the thoracic sympathetic nerve trunk, as previously described [17]. Inspiratory motoneuron activity was monitored through recordings from the ventral root of the fourth cervical spinal segment (C4). In brief, the vertebrae and left side of the thoracic cage were removed, and a part of the right side of the thoracic cage with the attached right side of the spinal nerve roots was retained to record the SNA from the right thoracic sympathetic nerve trunk (at the level of T10–T12) by a suction electrode. Preparations from younger rats (age: 0–1 days) were used for this experiment.
2. Spinal cord block preparation for membrane potential recordings from sympathetic preganglionic neurons in the IML and interneurons in the IML area. A spinal cord block including one or two segments of thoracic spinal cord (Th2-Th4) was dissected. The ipsilateral ventral nerve root was electrically stimulated (by 1–10 mV, 100 µs, single pulse) for confirmation of sympathetic preganglionic neurons [18].
3. Medulla-spinal cord preparation for membrane potential recordings from C1 area neurons in the RVLM. Inspiratory motoneuron activity was monitored at the ventral root of C4. C1 adrenergic neurons are characterized by the expression of tyrosine hydroxylase (TH) (or phenylethanolamine N-methyltransferase, PNMT) in the RVLM, some of which had descending projections to the IML in the spinal cord [20, 21]. However, it should be noted that some neurons projecting to the IML localizing closely to C1 adrenaline neurons were PNMT or TH negative [22]. Thus, the C1 area is a term referring to the region of the RVLM in which TH-positive and -negative neurons, including projecting neurons to the IML, exist and corresponds to the “sympathoexcitatory pressor area” of the RVLM [23–25]. We sought the C1 area neurons from the ventral side of the medulla. To confirm axonal projection to the IML of C1 area neurons, the ipsilateral IML at the Th2 level was electrically stimulated (by 1–10 mV, 100 µs, single pulse) using a tungsten electrode (30-μm tip diameter; Unique Medical, Tokyo, Japan) [26].

Nerve activity was recorded using an AC amplifier (MEG-5200, Nihon Kohden, Tokyo, Japan) through a 0.5-Hz low-cut filter and stored on hard-disk memory via a PowerLab system (ADInstruments, Castle Hill, Australia) with a sampling rate of 4 kHz.

### Drugs and solutions

Exendin-4 (a GLP-1 receptor agonist, Sigma-Aldrich, Tokyo, Japan) was stored as a 100 µM stock solution at −20 °C. We used 1–100 nM exendin-4 as previously described [27]. It was dissolved in artificial cerebrospinal fluid and bath applied. The GLP-1R antagonist 1 (compound 5 d, a GLP-1 receptor antagonist, MedChemExpress, NJ, USA) [4, 28] was stored as 5 mM stock solution at -20 °C. The antagonist was used at a concentration of 1 μM [28].

### Whole cell recordings

Intracellular recordings were performed using a blind whole-cell patch-clamp method [29, 30], with a high input impedance DC amplifier (CEZ-3100; Nihon Kohden). Electrodes were made by pulling thin-wall borosilicate glass (TW100F-4; World Precision Instruments, Inc. Sarasota, USA) while heating. The diameter of the inner tip was 1.2–2.0 μm, and the resistance was 4–8 MΩ. The electrodes were filled with a pipette solution (in mM) of 130 K-gluconate, 10 EGTA, 10 HEPES, 2 Na_2_-ATP, 1 CaCl_2_, 1 MgCl_2_, at pH7.2-7.3, adjusted by KOH. The electrode tip was filled with 0.2% Lucifer Yellow (lithium salt, Sigma-Aldrich) or 0.2% neurobiotin (Vector Lab., Inc., Burlingame, CA, USA) dissolved in the same solution used for the histological analysis of the recorded cells. The electrical signals were amplified (×10) and digitized at 4 kHz (PowerLab, ADInstruments), then stored on a personal computer using the LabChart 7 Pro software program (ADInstruments).

### Immunofluorescence

The samples were fixed in 4% paraformaldehyde in 0.1 M phosphate buffer solution at 4 °C and stored for histological analysis. Transverse 30- or 50-μm slices were cut with a vibrating-blade tissue slicer (PR07; Dosaka EM Co. Ltd., Osaka, Japan) or with a cryostat (CM1520; Leica Biosystems, Nussloch, Germany) for immunohistochemical examination. The following primary antibodies were used for immunofluorescence: rabbit anti-Lucifer yellow (1:500 dilution, Molecular Probes/Invitrogen), rabbit anti-GLP-1 receptors (ab218532, 1:1000 dilution, Abcam) and chicken anti-TH antibody (1:1000 dilution, Abcam). The secondary antibodies for fluorescence staining (1:1000 dilution) were Alexa Fluor 488 anti-rabbit IgG (Molecular Probes/Invitrogen), Alexa Fluor 546 or 633 anti-chicken IgG (Thermo Fisher Scientific/Invitrogen). For identification of GLP-1 receptor expression on recorded neurons, neurobiotin was used instead of Lucifer yellow to label cells. Neurobiotin-labeled neurons were visualized by incubation with avidin (Alexa Fluor 488 conjugate; 1:500 dilution, Thermo Fisher Scientific). DAPI (4’,6-diamidino-2-phenylindole, Sigma-Aldrich) was used for nuclear acid staining. The locations of the cell bodies of neurons recorded in the IML were confirmed after staining with NeuroTrace (435/455 blue or 530/615 red fluorescence, Invitrogen). Images of the immunofluorescent samples were obtained using an FV1000 confocal microscope (Olympus Optical, Tokyo, Japan) or conventional fluorescence microscope (BX60; Olympus Optical).

### Data analysis

All data analyses were performed using the LabChart 7 Pro software program (ADInstruments). The burst rate of C4 inspiratory activity was calculated as the mean of 10–20 respiratory cycles. The mean firing frequency of tonic firing neurons was calculated from an interval of 20 spikes. To measure the amplitude of SNA, the first derivative of the raw signals was rectified and integrated with a 20 s time constant in off-line analysis by the LabChart 7 Pro software program. Data are presented as the mean and standard deviation. Normality was assessed using the Kolmogorov–Smirnov test. Subsequently, the significance of the values in all cases was analyzed by one-way repeated measures analysis of variance followed by a Tukey–Kramer multiple comparisons test (GraphPad InStat; GraphPad Software Inc., La Jolla, CA, USA). Comparisons between neuron groups were performed using Student’s t-test, following confirmation of equal variances via an F-test using Excel for Microsoft Office 365. *P* values of < 0.05 were considered to indicate statistical significance. Graph charts for data plots were created using Origin 2020 (OriginLab Corporation, Northampton, MA 01060, USA).

## Results

### Effects of exendin-4 on SNA and C4 respiratory activity

**Fig. 1:**
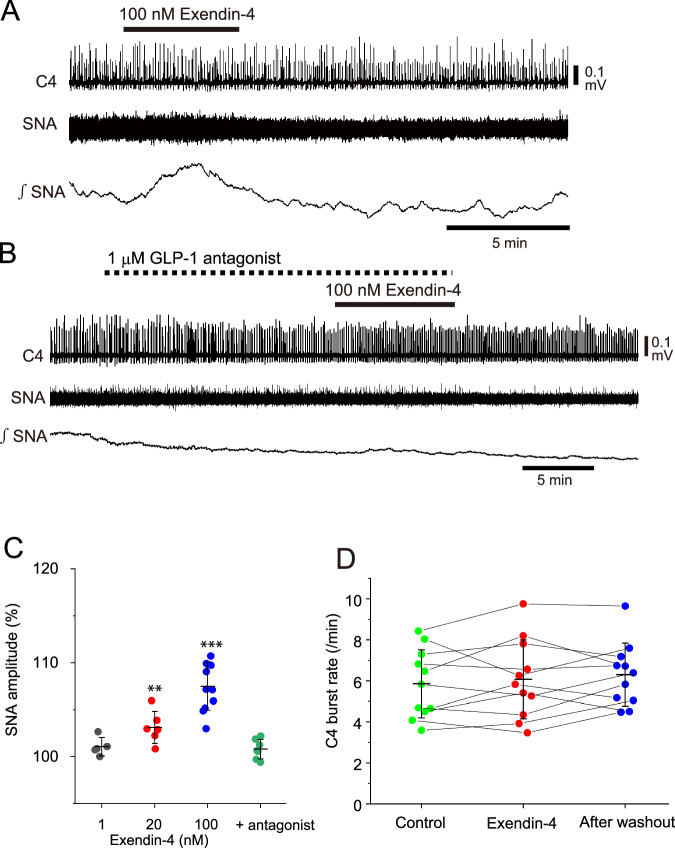
Effects of exendin-4 on sympathetic nerve activity (SNA) and C4 activity. **A** An example of C4 and SNA recordings when 100 nM exendin-4 was applied. Upper to lower traces: C4 activity, SNA and integrated SNA with a 20-s time constant. Exendin-4 induced an increase in the amplitude of the SNA that reached the peak value at 3 min after start of the application. **B** An example of the effects of 1 µM glucagon-like peptide-1 (GLP-1) antagonist (compound 5 d) on the SNA change induced by exendin-4. Note that the SNA trace indicates tendency of nonspecific decrease with time. The excitatory effect induced by exendin-4 was attenuated in the presence of the antagonist. **C** Data plots of changes (%) in the SNA amplitude measured at different doses. Black circles, peak values at 1 nM; red circles, values at 20 nM and blue circles, values at 100 nM exendin-4. Green circles denote values at 100 nM exendin-4 in the presence of 1 µM GLP-1 antagonist. Bars denote the mean ± standard deviation (SD). ** *P* < 0.01, *** *P* < 0.001, relative to control values, as determined by a one-way repeated measures analysis of variance followed by a Tukey-Kramer multiple comparisons test. **D** Data plots of the C4 burst rate. Green circles, values in control; red circles, 10-min 100 nM exendin-4; blue circles, 10 min after washout. Bars denote the mean ± SD. The burst rate did not change significantly

First, we examined the effects of different concentrations of exendin-4 (1, 20 and 100 nM) on SNA and C4 activity. Figure 1A shows a typical example of the effects of 100 nM exendin-4. During the application of 100 nM exendin-4, integrated SNA increased and typically reached the peak at 3–4 min after start of the application, followed with the decrease (Fig. 1A). Although 1 nM exendin-4 (*n* = 5) induced no significant change in SNA, concentrations of 20 nM (*n* = 6) and 100 nM (*n* = 11) significantly increased the peak amplitude of the SNA (Fig. 1C), indicating dose-dependent excitatory effects. The excitatory effect of exendin-4 was blocked by GLP-1 receptor antagonist 1 (compound 5 d) (10–15 min pretreatment of antagonist followed by 100 nM exendin-4 plus antagonist, *n* = 7) (Fig. 1B, C). In contrast, the C4 inspiratory burst rate did not change significantly with 100 nM exendin-4 (Fig. 1D).

### Effects of exendin-4 on IML neurons

**Fig. 2:**
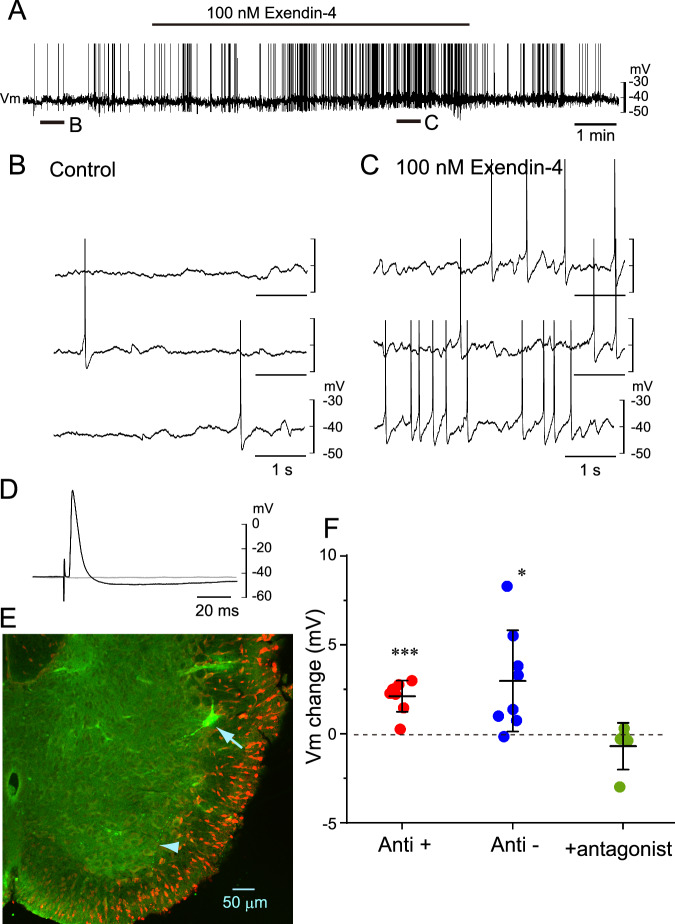
A typical example of the effects of exendin-4 on an intermediate lateral cell column (IML) neuron at the Th2 level. **A** Change in the membrane potential trajectory in response to the application of 100 nM exendin-4 to an IML neuron. **B** Faster sweep representation of membrane potential traces in control, corresponding to “B” in panel A. **C** Increased frequency of action potentials during 100 nM exendin-4 application, corresponding to “C” in panel **A**. **D** Antidromic action potential induced in an all-or-none fashion by electrical stimulation of the Th2 ventral root. **E** A Lucifer yellow-stained neuron merged with fluorescence Nissl staining by NeuroTrace (red). Note that the cell soma is located in the IML (an arrow) and an axon (arrowhead) has traveled toward the ventral root of Th2. **F** Data plots of the change in membrane potential (Vm) in response to 100 nM exendin-4. Red circles, antidromically activated cells stimulated by the corresponding Th ventral root; blue squares, non-antidromically stimulated cells. Dark green circles denote values at 100 nM exendin-4 in the presence of 1 µM glucagon-like peptide-1 antagonist. Bars denote the mean ± standard deviation. * *P* < 0.05, *** *P* < 0.001, as determined by a one-way repeated measures analysis of variance followed by a Tukey-Kramer multiple comparisons test

Next, we examined the effects of exendin-4 on IML neurons at the Th2–4 levels. Figure 2 shows a typical example of the effects of 100 nM exendin-4 on an IML neuron at the Th2 level. After the application of exendin-4, the cell was depolarized and the frequency of action potentials increased (Fig. 2ABC). This cell was confirmed to be a sympathetic preganglionic neuron by the antidromic action potential induced by stimulation of the Th2 ventral root (Fig. 2D). Figure 2E shows that this cell is located in the IML and an axon had traveled toward the ventral root of Th2. We analyzed the changes in membrane potential in antidromically activated preganglionic cells (*n* = 8) and interneurons (*n* = 8) that were not antidromically activated by ventral root stimulation. Figure 2F demonstrates that exendin-4 induced significant membrane depolarization in both types of cells. We also confirmed that the excitatory effect of exendin-4 on neurons in the IML region (4 preganglionic neurons and 1 interneurons) was blocked by GLP-1 receptor antagonist (10–15 min pretreatment of antagonist followed by 100 nM exendin-4 plus antagonist) (Fig. 2F).

### Effects of exendin-4 on C1 area neurons

**Fig. 3:**
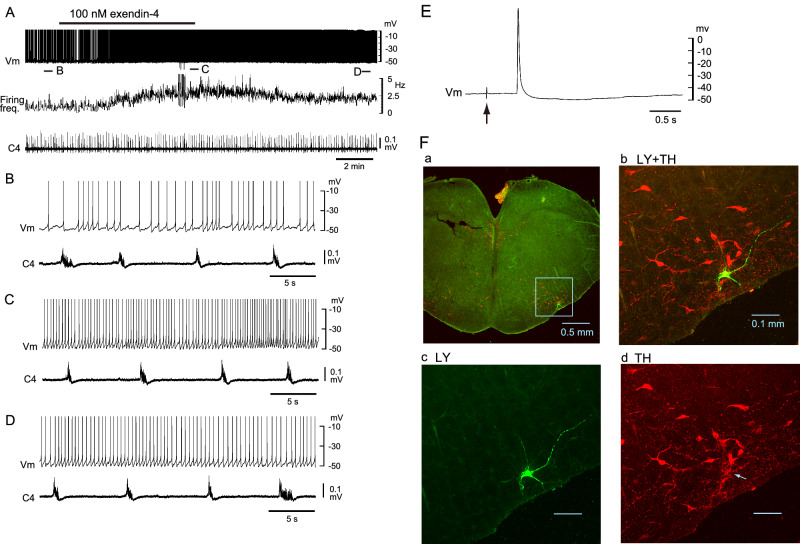
An example of TH-positive C1 area neurons. **A** Slower time course representation of membrane potential trajectory (Vm), firing frequency of action potentials and C4 inspiratory activity (C4). 100 nM exendin-4 enhanced the firing. B–D. Faster sweep representation of the traces corresponding to** B, C and D** in panel **A**. **E** This cell was antidromically activated by stimulation of the ipsilateral intermediate lateral cell column (IML) at the Th2 level. **F** Upper left panel **A**, location of the Lucifer yellow (LY)-labeled neuron recorded. Upper right **B** and lower panels **C, D** higher magnification views of the highlighted square in the left panel. LY (green), tyrosine hydroxylase (TH) (red). This neuron was TH-positive

**Fig. 4:**
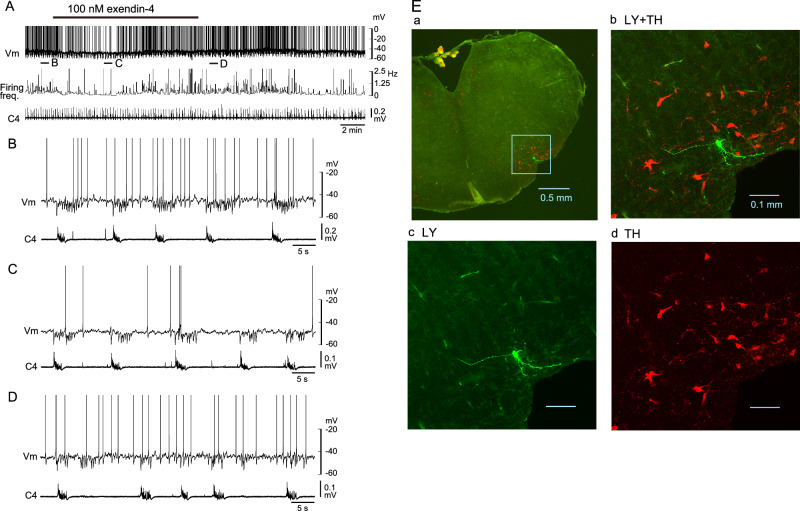
An example of C1 area neurons showing membrane hyperpolarization in response to exendin-4. **A** Slower time course representation of the membrane potential trajectory (Vm), firing frequency of action potentials and C4 inspiratory activity (C4). 100 nM exendin-4 initially induced membrane hyperpolarization and a decrease in firing frequency, followed by depolarization and an increase in firing frequency. This cell was not antidromically activated by stimulation of the ipsilateral intermediate lateral cell column (IML) at the Th2 level. **B–D** Faster sweep representation of the traces corresponding to **B, C and D** in panel **A**. **E** Upper left panel **A**, location of the recorded Lucifer yellow (LY)-labeled neuron. Upper right **B** and lower panels **C, D**, higher magnification views of the highlighted square in left panel. LY (green), tyrosine hydroxylase (TH) (red). This neuron was TH-negative

**Fig. 5:**
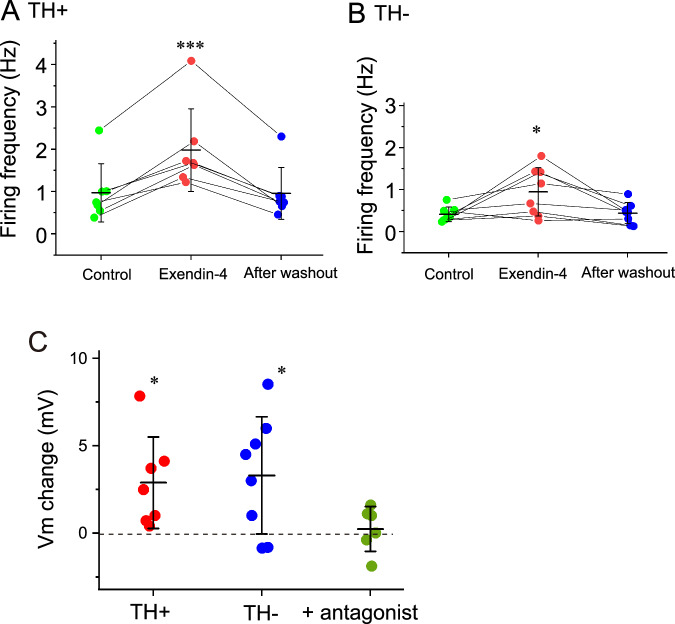
Data plots of the firing frequency and membrane potential change of rostral ventrolateral medulla neurons. **A** Tyrosine hydroxylase (TH)-positive C1 neurons. **B** TH-negative C1 area neurons. Green circles, firing frequency in control; red circles, during 100 nM exendin-4 (10 min); blue circles, after washout (10 min). **C** Data plots of the change in membrane potential (Vm) in response to 100 nM exendin-4. Red circles, TH-positive neurons; blue circles, TH-negative neurons. Dark green circles denote values at 100 nM exendin-4 in the presence of 1 µM glucagon-like peptide-1 antagonist. Bars denote the mean ± standard deviation. * *P* < 0.05, *** *P* < 0.001,asdetermined by a one-way repeated measures analysis of variance followed by a Tukey-Kramer multiple comparisons test

Finaly, we investigated the effects of exendin-4 on C1 area neurons in the RVLM. Neurons were categorized into two subtypes: (1) C1 adrenergic neurons (TH-positive tonic firing) and (2) other C1 area neurons (TH-negative tonic firing). Figure 3 shows an example of a TH-positive C1 neuron. The application of 100 nM exendin-4 induced an increase in firing frequency with slight membrane depolarization (Fig. 3BCD). This cell was antidromically activated by stimulation of the ipsilateral IML at the Th2 level (Fig. 3E) and was TH-positive (Fig. 3F). Figure 4 shows an example of TH-negative C1 area neurons. This cell showed initial hyperpolarization (Fig. 4C) followed by depolarization and an increase in firing frequency (Fig. 4D) in response to the application of 100 nM exendin-4. This cell was located in the C1 area but was TH-negative (Fig. 4E). The average firing frequency change of TH-positive (*n* = 7) and TH-negative (*n* = 8) neurons significantly increased during the application of 100 nM exendin-4 (Fig. 5AB), accompanied by significant membrane depolarization (Fig. 5C). A total of 4 neurons (one TH-positive and three TH-negative) exhibited initial transient hyperpolarization as observed in the cell shown in Fig. 4. We also confirmed that the excitatory effect of exendin-4 on C1 area neurons (4 TH-positive and 2 TH-negative) was blocked by GLP-1 receptor antagonist (10–15 min pretreatment of antagonist followed by 100 nM exendin-4 plus antagonist) (Fig. 5C). In the present study, 58% of TH-positive neurons and 33% of TH-negative neurons were antidromically activated by stimulation of the ipsilateral IML at the Th2 level. The antidromic latency was 39.1 ± 9.6 ms (*n* = 10). The firing of 67% of the above C1 area neurons indicated weak respiratory (typically expiratory) modulation [26], as seen in the cell in Fig. 4.

### Immunohistochemical examination of GLP-1 receptors

The GLP-1 receptor expression levels in the neurons of the RVLM and IML were reported in previous studies [16, 31] (see also discussion). We also confirmed these previous observations by an immunohistochemical examination. Supplementary Fig. S1 shows most TH-positive cells and many TH-negative cells in C1 area of the RVLM expressed GLP-1 receptors. In addition, we confirmed that cells in the RVLM (two TH-positive and two TH-negative cells that showed an excitatory response (3.2 ± 2.1 mV depolarization) to 100 nM exendin-4 application) expressed GLP-1 receptors (Supplementary Figs. S2 and S3). The expression of GLP-1 receptors was also detected in the IML at Th2–4 level (Supplementary Fig. S4). Moreover, we confirmed that neurons in the IML (two preganglionic neurons and two interneurons that showed an excitatory response (3.6 ± 1.4 mV depolarization) to 100 nM exendin-4 application) expressed GLP-1 receptors (Supplementary Fig. S4 and S5).

## Discussion

### Effects of excendin-4 on sympathetic nerve activity and neurons of the intermediate lateral cell column and rostral ventrolateral medulla

We found that exendin-4 induced increases in SNA and firing rates of the preganglionic IML neurons and interneurons in the IML region, accompanied by significant membrane depolarization. In addition, exendin-4 significantly increased the firing rates of TH-positive and TH-negative C1 area neurons in the RVLM, accompanied by membrane depolarization. The excitatory effects of exendin-4 on these sympathetic neuron activities were blocked by GLP-1 receptor antagonist, suggesting that the effects were due to the activation of GLP-1 receptors by exendin-4. Some C1 area (TH-positive and -negative) neurons were confirmed to be bulbospinal presympathetic neurons because they were antidromically activated by ipsilateral IML stimulation, which is basically consistent with the results of previous studies [26, 32–34].

Exendin-4 exerted excitatory effects on SNA at concentrations greater than 20 nM. The dose dependency in our results was consistent with a previous study by Wan et al. [27], who demonstrated a similar effective range (10–1000 nM) in preganglionic vagal motoneurons of slice preparations. In most cases of our experiments, the peak SNA activity during exendin-4 application appeared before washout (Fig. 1A). This might be attributable to desensitization of GLP-1 receptors [35, 36]. Exendin-4 seemed to pass the blood–brain barrier [37–39], and the typical plasma concentration of GLP-1 receptor agonists in clinical practice is estimated to be approximately 0.1–0.3 nM/L. Although the 100 nM concentration used in this study is substantially higher, Wan et al. [27] discussed differences in effective concentrations between experimental conditions (i.e., the concentration–response curve to agonists shifted to the right by two or more orders of magnitude and the GLP-1 effects in the nanomolar range were within the acceptable physiological range). On the other hand, Oshima et al. [16] showed that C1 adrenergic neurons in the RVLM were hyperpolarized by a low concentration (0.2 nM) of GLP-1 peptide. This effect implies that endogenous GLP-1 may induce inhibitory effects on RVLM neurons, resulting in a decrease in SNA. In the present study, we did not detect depressive effects of low-concentration (1 nM) exendin-4 on SNA. However, some C1 area neurons showed initial hyperpolarization followed by depolarization during the application of 100 nM exendin-4. The initial response might reflect the inhibitory effect of activation of GLP-1 receptors, as suggested by Oshima et al. [16].

### Locations of GLP-1 receptors in the medulla and spinal cord

Many previous studies have reported the sites of GLP-1 receptor expression in various brain regions, including the brainstem, diencephalon, and cerebrum [3, 4, 40–42]. In the medulla, the expression of GLP-1 receptors was found in C1 neurons of the RVLM [16] and in neurons of the NTS [2, 41]. The IML neurons (i.e., sympathetic preganglionic neurons) at the Th1-2 levels as well as the interneurons around the IML also expressed GLP-1 receptors [31]. We also confirmed the GLP-1 receptor expression in RVLM and IML neurons in the present study. Therefore, it was considered that there are at least three possible pathways for the activation of sympathetic preganglionic neurons by exendin-4 under the present experimental conditions: a direct postsynaptic effect, an indirect effect via interneurons in the spinal cord, and descending excitation by bulbospinal (presympathetic) C1 area neurons in the medulla.

### Effects of GLP-1 receptor agonists on the sympathetic nervous system

GLP-1 receptor agonists could induce an increase of heart rate, as reviewed by Lorenz et al. [5], possibly due to activation of cardiac sympathetic preganglionic neurons, as described by Holt et al. [2] Smits et al. [14] suggested that exenatide-induced tachycardia involves activation of the sympathetic nervous system in humans. Furthermore, Osaka et al. [43] suggested that GLP-1 might play a role in postprandial energy expenditure mediated by the lower brainstem and the sympathoadrenal system. Recently, Xu et al. [4] demonstrated that the activation of GLP-1 receptors in the hypothalamic paraventricular nucleus increased SNA. Our findings suggest that GLP-1 receptor activation in the medulla and spinal cord also induces an increase in SNA in in vitro preparations, thereby providing additional evidence to support previous findings.

### Other mechanisms related to tachycardia

Several other mechanisms for the induction of tachycardia by GLP-1 agonists have been proposed. The first is the suppression of parasympathetic activity by exendin-4, which inhibits neurotransmission to cardiac vagal neurons in the nucleus ambiguus [44]. The second is reflex tachycardia in response to vasodilation [45]. However, Smits et al. [14] reported that exenatide did not alter total peripheral resistance or diastolic blood pressure, which does not support the reflex tachycardia hypothesis. The third mechanism involves the presence of GLP-1 receptors in the sinoatrial node [46, 47], and it is proposed that GLP-1 receptor agonists may directly act on the sinoatrial node to induce tachycardia [48]. However, these reports do not negate the involvement of the effects of GLP-1 agonists on the sympathetic nervous system, and it is plausible that tachycardia is induced through multiple mechanisms.

### Other effects of GLP-1 receptor agonists

Although previous studies reported that GLP-1 agonists induced an increase in blood pressure as an acute response, mediated by activation of the sympathetic nervous system [4, 49], it was also noted—especially as a long-range effect in clinical setting—that these drugs could decrease blood pressure, as described in multiple reviews [50–53]. The blood pressure decrease might be due to indirect effects such as diuretic action [54] or a reduction in angiotensin II (Ang II) concentrations [55]. Thus, blood pressure might be influenced via GLP-1 receptors by multiple mechanisms, as reviewed by Goud et al. [56].

Regarding the effects on respiration, no effect of exendin-4 was observed in the present study. Although exendin-4 has been reported to improve chronic obstructive pulmonary disease in clinical settings [57, 58], the mechanisms are related to immune-modulating or anti-inflammatory effects, and there are no reports on its direct effects on respiratory neuron activity.

## Limitations

We used in vitro preparations from newborn rats (P0–P4, either sex) in the present experiment. Although sex difference is an important factor in the actions of GLP-1 and its analogs [59], the present study provides no information about this issue. Therefore, the results should be compared carefully with those from older rats (in vivo) or humans. It has been reported that glucose concentration affects RVLM neuron activity [16]. In the present study, the glucose concentration was kept at 30 mM, which was the standard concentration in the experiment, using in vitro preparation from newborn rats and we did not test the effects of different glucose concentrations on SNA. The blood pressure could not be recorded because extracellular solution is applied by superfusion.

## Conclusion

Our study is the first to demonstrate a direct link between GLP-1 receptor agonists and sympathetic-related neurons in the spinal cord and medulla. This finding provides a clear basis for the established acute effects of GLP-1 receptor agonists inducing increases in blood pressure, heart rate, and SNA. Our findings offer critical insights into the cardiovascular actions of GLP-1 receptor agonists and may inform safer therapeutic strategies for patients with type 2 diabetes.

## Supplementary information

Supplementary figures
