# Supplementary material for: The effects of glucagon-like peptide-1 receptor agonists on sympathetic neuron activity
Source: Hypertens Res. 2026 Apr 10;49(6):1939–50. doi: 10.1038/s41440-026-02633-5 (PMC13236593; doi:10.1038/s41440-026-02633-5)
Supplement: Supplementary file 1 — Supplementary figures [file 41440_2026_2633_MOESM1_ESM.pdf]

Suppl. Fig. S1

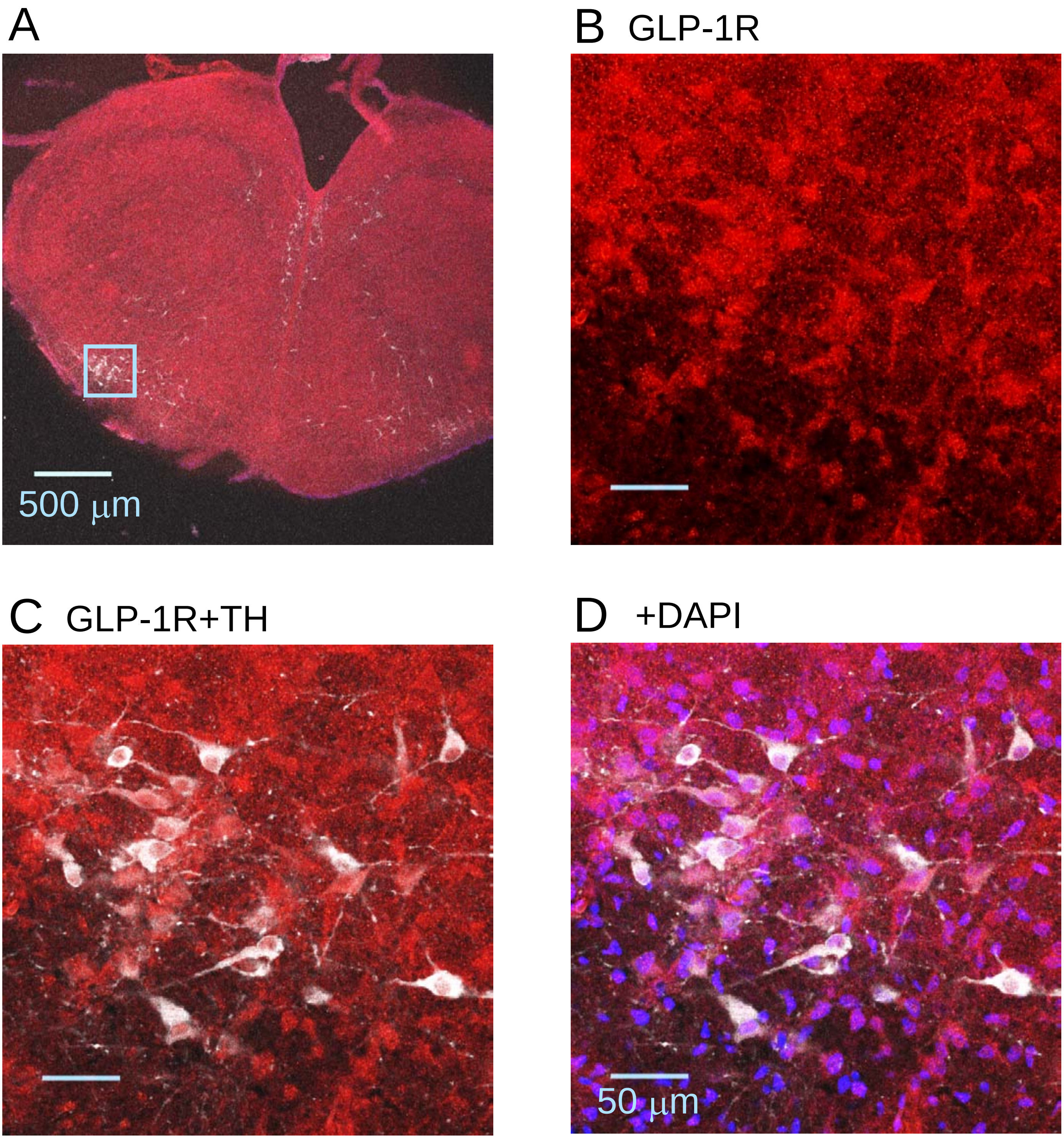

The glucagon-like peptide-1 receptor (GLP-1R) expression in the C1 area of the rostral ventrolateral medulla (RVLM). A. Lower magnification view of the RVLM. B–D. Higher magnification views of the highlighted square in A. B. GLP-1R (red). C. GLP-1R+tyrosine hydroxylase (TH; white). D. GLP-1R+TH+4',6 diamidino-2-phenylindole (DAPI; dark blue). Note that most of the TH-positive cells and many TH-negative cells expressed GLP-1R.

Suppl. Fig. S2

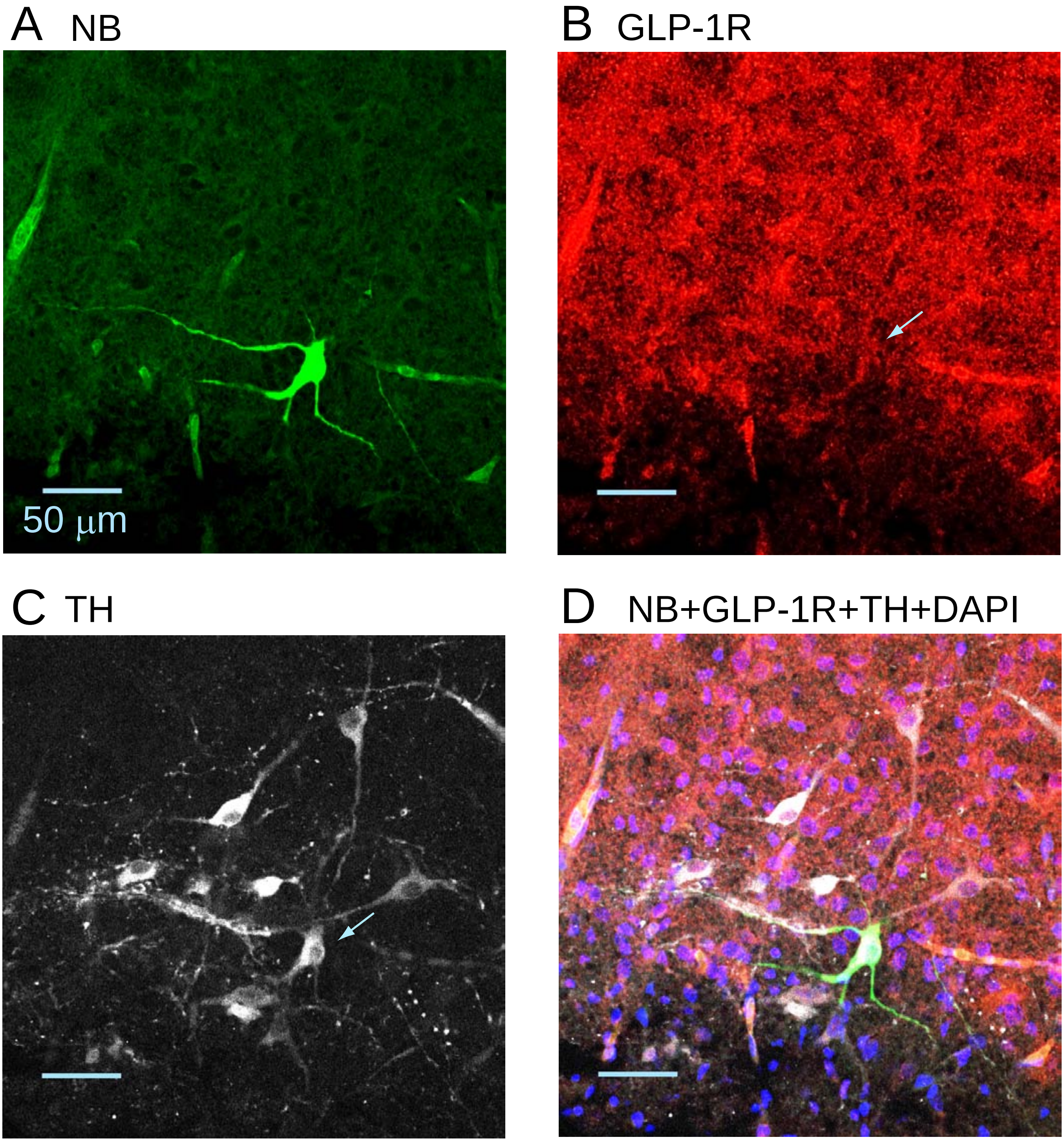

An example of a neurobiotin (NB)-labeled (TH-positive) neuron in the C1 area that showed an excitatory response to the application of 100 nM exendin-4. A. An NB-labeled neuron (green). B. Glucagon-like peptide-1 receptor (GLP-1R; red). C. Tyrosine hydroxylase (TH; white). D. NB+GLP-1R+TH+4',6 diamidino-2-phenylindole (DAPI). Note that this cell expresses GLP-1R (B, arrow) and is TH-positive (C, arrow).

Suppl. Fig. S3

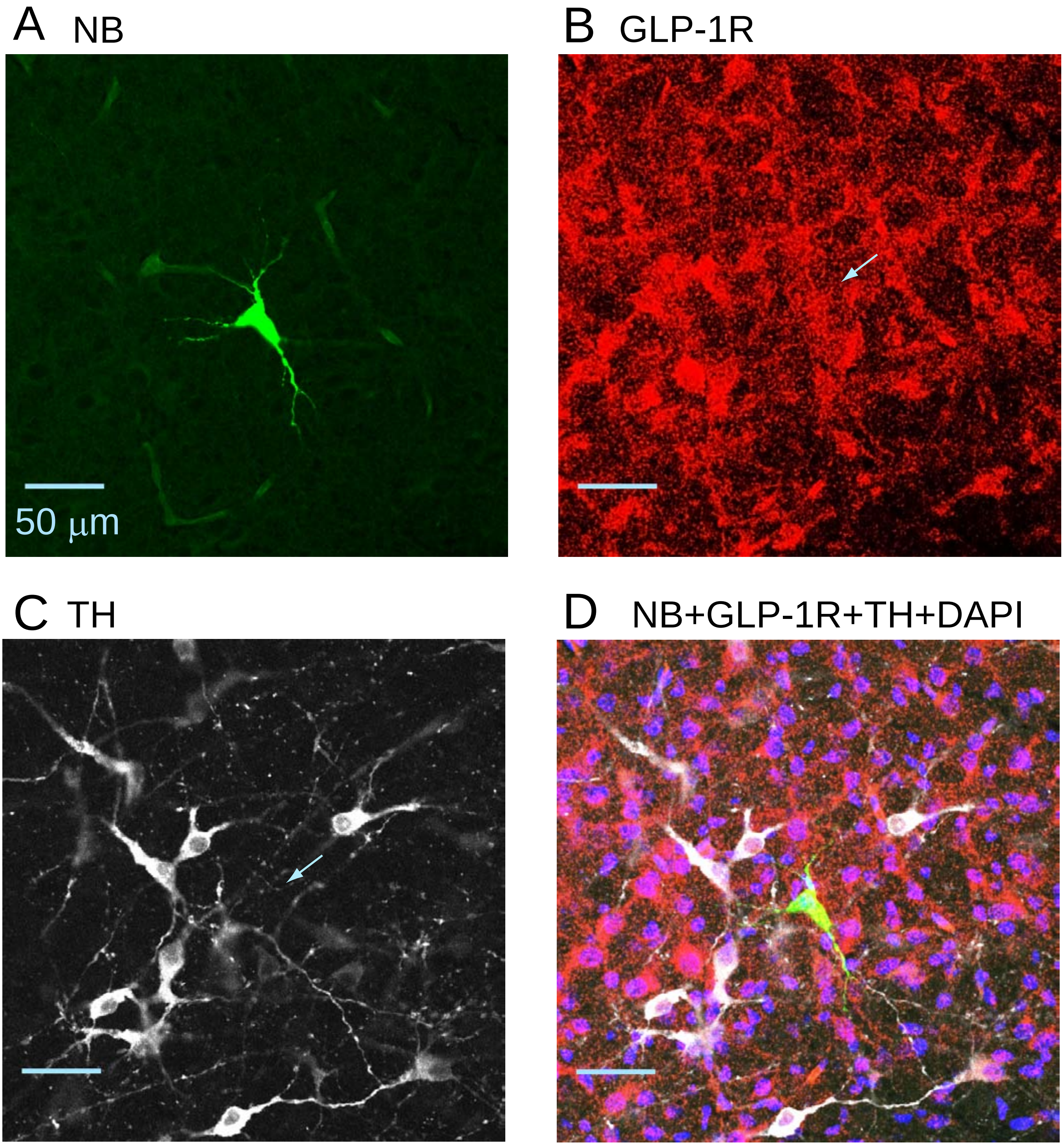

An example of a neurobiotin (NB)-labeled (TH-negative) neuron in the C1 area that showed an excitatory response to the application of 100 nM exendin-4. A. An NB-labeled neuron (green). B. Glucagon-like peptide-1 receptor (GLP-1R; red). C. Tyrosine hydroxylase (TH; white). D. NB+GLP-1R+TH+4',6 diamidino-2-phenylindole (DAPI). Note that this cell expresses GLP-1R (B, arrow) and is TH-negative (C, arrow).

Suppl. Fig. S4

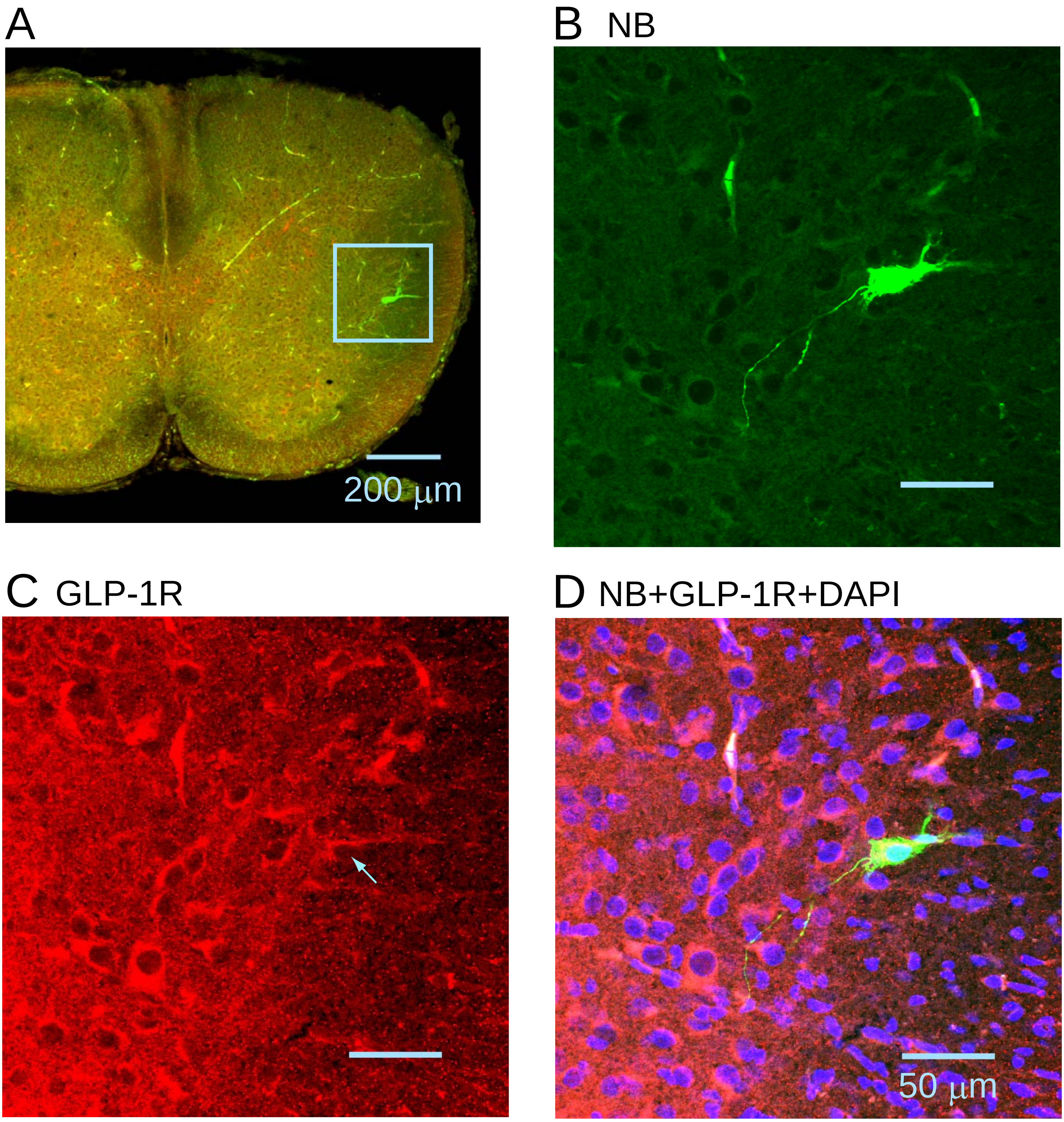

The glucagon-like peptide-1 receptor (GLP-1R) expression in the intermediate lateral cell column (IML) at the Th2 level and an example of a neurobiotin (NB)-labeled neuron that showed an excitatory response to the application of 100 nM exendin-4. This cell was identified to be a sympathetic preganglionic neuron because it was antidromically activated by ventral root stimulation. A. Lower magnification view of the spinal cord slice at the Th2 level. B–D. Higher magnification views of the IML region (highlighted square in A). B. NB (green). C. GLP-1R (red). D. NB+GLP-1R+4',6 diamidino-2-phenylindole (DAPI). Note the expression of GLP-1R in cells of the IML region, including the recorded cell (C, arrow).

Suppl. Fig. S5

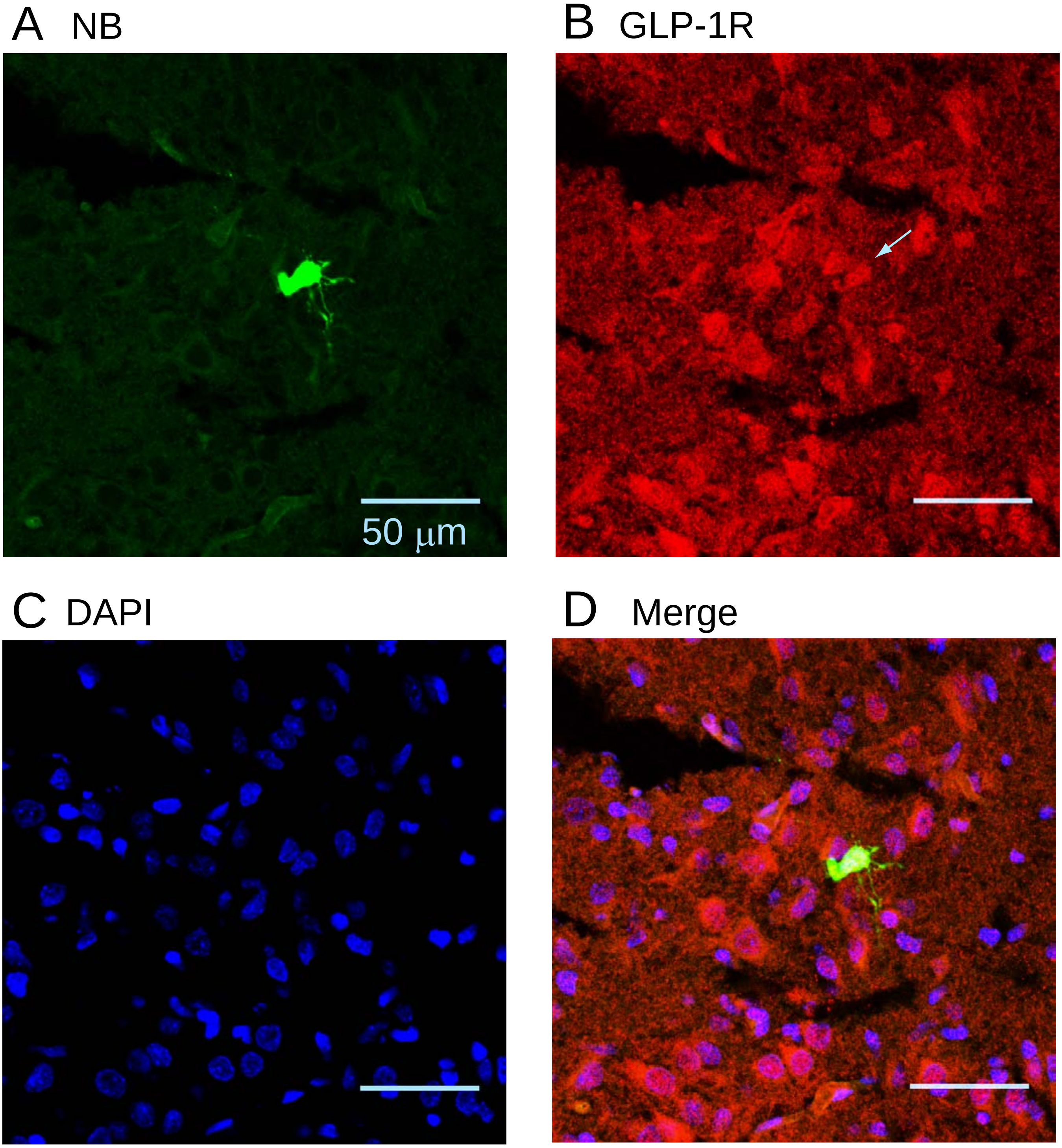

An example of a neurobiotin (NB)-labeled neuron in the intermediate lateral cell column area at the Th 3 level that showed an excitatory response to the application of 100 nM exendin-4. This cell was thought to be an interneuron because it was not antidromically activated by ventral root stimulation. A. An NB-labeled neuron (green). B. Glucagon-like peptide-1 receptor (GLP-1R; red). C. 4',6 diamidino-2-phenylindole (DAPI; dark blue). D. Merge. Note that this cell expresses GLP-1R (B, arrow).
